# Effects of Climate on Northern and Southern Constitutions

**Published:** 1861-08

**Authors:** 


### EFFECTS OF CLIMATE ON NORTHERN AND SOUTHERN CONSTITUTIONS.

Public health is public wealth. This short aphorism has, with the advance of sanitary science, acquired a meaning which political economists of the last century hardly dreamed of. But the preservation of the public health in time of peace is not more important to the State than is the sanitary condition of its armies in time of war. The health of an army is the strength thereof. A recognition of this fact by the Federal Government is apparent in the appointment of a Sanitary Commission, whose duty it shall be to establish a proper surveillance over the troops in camp and in the field, to adopt such measures as will place them in the best condition to resist the influences which affect their health, to see that their food, clothing, &c., are proper and sufficient, and that nothing shall be present to weaken the efficiency of the force from causes affecting the health of our soldiers.

The effects of a Southern climate upon the Northern constitution is a subject which will particularly attract the attention of this Commission. Whatever these effects may be, they can be mitigated in a great measure by the acts of this Commission, the concerted aid of the medical officer of the corps, and the intelligent understanding of the subject by the soldier himself.

Comparing the Northern soldier with the Southern, we believe the former will withstand the effects of the climate for a short campaign of a year or more better than the latter; and though the popular belief is divergent to this view, the statistics of our war with Mexico fully sustain it, and the published opinion of no less an authority than Dr. Nott, of Mobile, in the *Southern Journ. of Med. and Pharmacy*, for January, 1847, confirms it.

The statistics of the Mexican War are so remarkable, that we present them as we find them, given in a recent number of the *Evening Post*:

On April 8th, 1848, the Secretary of War made a report to the United States Senate of the losses of the volunteer forces employed in Mexico. From this, it appears that seven Northern States—Massachusetts, New York, New Jersey, Pennsylvania, Ohio, Indiana and Illinois—furnished, in the course of that war, 22,573 men. Of this force, the total loss from disease was 2,931 men; less than one-eighth of the whole. Nine slave States—Virginia, North Carolina, South Carolina, Georgia, Alabama, Mississippi, Louisiana, Tennessee and Kentucky—furnished 22,899 men. The loss from this force by disease, was 4,315, or more than one-fifth; a very considerable difference in favor of Northern troops.

When we go into particulars, we find that Massachusetts lost, of 1,047 men, but 61 by disease; while South Carolina, furnishing 1,054 men, or seven more than Massachusetts, lost not less than 338 by disease. Mississippi lost 769 men by disease, out of 2,319; while Indiana, furnishing nearly double the number, namely, 4,470, lost only 768. Georgia lost 362 men by disease, out of 2,047, while New York lost but 188 of a total of 2,665. North Carolina sent 936, and lost 233; while New Jersey sent 424, and lost but 12. Pennsylvania sent 2,464, and lost 411; Mississippi lost 769 out of 2,319. Illinois furnished 5,973 men, and lost 850; while Tennessee furnished only 3,011; Arkansas, 136 of a force of 1,323; rather more than ten per cent. in each. Kentucky lost 709 out of 4,800; but Ohio, sending 5,530, lost but 642. The Texan troops, fighting in a country to whose circumstances and climate they were thoroughly accustomed, lost yet more by disease than the Missourians, who came there from the cold North. Of 7,313 men, Texas lost 360 by disease; while of 6,753 Missourians, only 342 were thus lost.—*Am. Med. Monthly.*

